# Impact of inflammation and the immune system on hepatocellular carcinoma recurrence after hepatectomy

**DOI:** 10.1002/cam4.7018

**Published:** 2024-03-08

**Authors:** Sha She, Jinzhi Shi, Jiling Zhu, Fan Yang, Jia Yu, Kai Dai

**Affiliations:** ^1^ Department of Infectious Diseases Renmin Hospital of Wuhan University Wuhan Hubei Province China; ^2^ Department of Hepatobiliary surgery Renmin Hospital of Wuhan University Wuhan Hubei Province China

**Keywords:** hepatectomy, hepatocellular carcinoma, immune, inflammatory, recurrence

## Abstract

Hepatocellular carcinoma (HCC) is one of the leading causes of cancer‐related death worldwide. Hepatectomy remains the first‐line treatment for patients with resectable HCC. However, the reported recurrence rate of HCC at 5 years after surgery is between 50% and 70%. Tumor‐related factors, including tumor size, number and differentiation, and underlying liver disease are well‐known risk factors for recurrence after treatment. In addition to tumor‐related factors, ever‐increasing amounts of studies are finding that the tumor microenvironment also plays an important role in the recurrence of HCC, including systemic inflammatory response and immune regulation. Based on this, some inflammatory and immune markers were used in predicting postoperative cancer recurrence. These include neutrophil‐to‐lymphocyte ratio, platelet‐to‐lymphocyte ratio, cytotoxic T cells, and regulatory T cells, among others. In this review, we summarized the inflammatory and immune markers that affect recurrence after HCC resection in order to provide direction for adjuvant therapy after HCC resection and ultimately achieve the goal of reducing recurrence.

## 1 INTRODUCTION

Global Cancer Statistics 2020 reported that hepatocellular carcinoma (HCC) is the sixth most commonly diagnosed cancer worldwide and the third leading cause of cancer‐related death.^1^ In China, HCC is the second most common malignancy, and an estimated 392,868 new HCC cases, including 368,960 deaths, were reported as of 2018.^2^ At present, hepatectomy is a common and safe treatment option for patients with early‐stage HCC. Unfortunately, the 5‐year recurrence rate after resection is as high as 50%–70%; approximately 50% of HCC patients experience recurrence within 2 years.^3^, ^4^ Therefore, it is crucial to identify and characterize influencing factors of recurrence as well as patients who are at high risk of recurrence after liver resection.

Several major risk factors have been identified for HCC recurrence after surgery, including tumor size,^5^ number of nodules,^6^ degree of differentiation,^7^ vascular invasion,^8^ resection margin,^9^ and liver capsule invasion.^10^ It has been reported that systemic inflammation and immunity play a crucial role in the occurrence, metastasis, and recurrence of cancers, including HCC.^11^ In the past 10 years, a large number of clinical cohort studies have constructed models of inflammation, immunity, and inflammation‐immunity to predict the prognosis and recurrence of HCC after resection.^12^, ^13^, ^14^

In this review, we discuss inflammatory and immune markers associated with prognosis and recurrence after HCC resection as well as the mechanisms that influence recurrence. The predictive values of various inflammatory and immune‐related models are summarized to provide a basis for identifying HCC subsets with high recurrence risk. Moreover, we provide some direction for the adjuvant treatment of hepatectomy.

## 2 RISK FACTORS OF RECURRENCE AFTER CURATIVE HEPATECTOMY FOR HCC

According to the 2022 edition of the “Guidelines for the Diagnosis and Treatment of Primary Liver Cancer” in China, the indications for HCC resection range from Ia to IIIa and Ib, which correspond to stage A, B, and C of Barcelona Clinic Liver Cancer staging. However, the overall survival (OS) of patients after resection of HCC remains poor. Due to the different recurrence etiology, many clinical studies classify HCC resection into early recurrence and late recurrence. Early recurrence, defined as occurring within 2 years of initial treatment, is thought to be caused by intrahepatic metastases,^15^ whereas late recurrence occurs through de novo tumorigenesis or multicentric HCC associated with background liver disease such as hepatitis and/or cirrhosis.^16^

Large number of studies have shown that the prognosis and survival time of late recurrence after hepatectomy were significantly better than that of early recurrence. It was found that the different risk factors led to the difference in prognosis of recurrence type. Previous studies have also shown that the main risk factors for early recurrence are preoperative hepatitis B e antigen positivity, α‐fetoprotein (AFP) level above 400 ng/mL, age, preoperative liver function, and pathological characteristics of the tumor, including number, size, stage, vascular invasion, surgical margin (<2 cm), and pathological differentiation.^17^, ^18^, ^19^, ^20^, ^21^ Risk factors for late recurrence were higher hepatitis activity, liver cirrhosis, gross tumor type, and sex (being more common in men).^21^, ^22^ However, these risk factors are difficult to monitor in daily clinical work, and the sensitivity and specificity of AFP levels in monitoring HCC recurrence are low. Therefore, it is necessary to find new combined prognostic indicators to predict HCC recurrence after hepatectomy.

It is well known that systemic inflammation and immunity play crucial roles in cancer initiation and metastasis. Studies in the past 10 years have confirmed that systemic inflammatory and immune indicators can predict the prognosis and postoperative recurrence of various tumors, including those of liver cancer. Hashimoto et al.^23^ found that elevated preoperative C‐reactive protein is a poor prognostic factor for patients undergoing hepatectomy for HCC. Interleukin‐6, as one of the major regulators of C‐reactive protein production, has also been confirmed to be involved in metastasis and recurrence of liver cancer.^24^ Moreover, various inflammatory cells such as neutrophils, platelets, and monocytes have also been shown to be associated with the prognosis of HCC after resection.^12^, ^25^, ^26^

The tumor microenvironment plays a crucial role in promoting the progression and escape of tumor cells to form metastases. Recent studies have focused on tumor immune cell infiltration to predict the outcome after surgical treatment and construct immune scoring systems. Studies have found that lymphocyte subsets (CD3+, CD4+, CD8+, regulatory T (Treg) cells, etc), B cells, and natural killer (NK) cells are associated with OS, disease‐free survival (DFS), and relapse‐free survival after hepatectomy.^13^, ^27^, ^28^, ^29^ Based on this, this paper will analyze the influencing factors of recurrence after hepatectomy from the perspective of inflammatory immune microenvironment.

## 3 ROLE OF INFLAMMATION IN RECURRENCE OF HCC AFTER CURATIVE RESECTION

### 3.1 Glasgow prognostic score

Evaluation via the inflammation‐based Glasgow prognostic score (GPS), derived from standard thresholds of C‐reactive protein and albumin, can reflect the systemic inflammatory response and immune status of tumor patients. Patients were classified into three groups: patients with normal albumin (≥3.5 g/dL) and normal CRP (≤1.0 mg/dL) as GPS 0, those with low albumin (<3.5 g/dL) or elevated CRP (>1.0 mg/dL) as GPS 1, and both low albumin (<3.5 g/dL) and elevated CRP (>1.0 mg/dL) as GPS 2. Recent studies have found the GPS to be a clinically useful scoring system for predicting the prognosis of patients with various tumors after resection, including esophageal cancer,^30^ pancreatic cancer,^31^ colorectal cancer,^32^ etc.

Several studies have also investigated the relationship between GPS and the recurrence and prognosis of HCC patients after hepatectomy. A study from Japan was the first to demonstrate that GPS can effectively predict the survival of patients after resection of HCC by retrospectively analyzing the relationship between the survival time and GPS score of 398 patients after liver resection.^33^ Pan et al.^34^ found that the 3‐year and 5‐year recurrence probabilities of patients with GPS of 0 were 47.0% and 71.2%, respectively. Among patients with a GPS of 1 or 2, these rates were 76.7% and 100%, respectively.

The latest study found that the modified GPS (also named the inflammation based index (IBI)—which combines elevated CRP (>1 mg/dL) and hypo‐albuminaemia (<35 g/L)) is an independent predictor of DFS after resection of HCC and that it is more suitable than the original GPS to predict recurrence after hepatectomy.^35^ Collectively, all these studies suggest that GPS or the modified GPS can effectively predict recurrence in HCC patients after hepatectomy. However, each of these studies was limited by that fact that impaired liver function in patients with HCC may affect GPS and lead to biased prediction. This suggests that other causes of abnormal liver function should be excluded in the prognosis assessment and follow‐up of patients after hepatectomy. And the same patient may require multiple models of prediction to provide optimal prevention.

#### 3.1.1 Albumin‐bilirubin (ALBI) grade

Albumin‐bilirubin (ALBI) score was originally developed as an evidence‐based scoring system specifically designed to assess liver function in HCC.^36^ Subsequent studies have found that ALBI grading is associated with survival and tumor recurrence after hepatectomy.^37^, ^38^ ALBI score only uses albumin and bilirubin in a complex nomogram ([log10 bilirubin (in lmol/L) × 0.66] + [albumin (in g/L) × −0.085]), such that grades 1, 2, 3 = ≤−2.60, <−2.60 to ≤−1.39, >−1.39, respectively. Both preoperative and postoperative ALBI grades were found to be independently associated with postoperative recurrence. Lee's study found that preoperative ALBI grading, but not postoperative ALBI, was an independent risk factor for early recurrence (1 year).^39^ Two retrospective studies from Taiwan have demonstrated a more effective role of postoperative than preoperative ALBI grade in predicting late‐recurrences and long‐term prognosis, even years after surgery.^40^ Moreover, ALBI grading was a better predictor of postoperative outcomes and major complication.^40^ ALBI grade also predicts survival, toxicity and post‐procedural liver failure in patients treated with transarterial chemoembolisation, radioembolisation, external beam radiotherapy as well as multi‐kinase inhibitors (sorafenib, lenvatinib, cabozantinib, and regorafenib) and immune checkpoint inhibitor therapy.^40^ Based on these, ALBI grade has the potential to be used as an additional tool for selecting surgical candidates, and guiding physician and patient decisions.

### 3.2 Neutrophil‐to‐lymphocyte ratio and platelet‐to‐lymphocyte ratio

Emerging evidence has implicated host inflammatory responses in cancer progression and patient survival and characterized it as an independent prognostic factor for various types of cancer. The host response in systemic inflammation is associated with a disturbance of various blood components, such as leukocytes (especially neutrophils, lymphocytes, and monocytes) and platelets. Several studies have verified that the neutrophil‐to‐lymphocyte ratio (NLR), platelet‐to‐lymphocyte ratio (PLR), and lymphocyte‐to‐monocyte ratio (LMR) are predictors of HCC recurrence.^25^, ^41^

In a series of 958 patients, those with high preoperative NLR (≥2.81) had a higher recurrence rate after surgery compared with patients with low NLR levels.^42^ Lu et al.^43^ found that preoperative NLR >2.81 was significantly associated with tumor recurrence in resection patients with the Barcelona Clinic Liver Cancer stage 0/A or B HCC (*p* < 0.05). Another study also confirmed that preoperative NLR was associated with early tumor recurrence in patients undergoing hepatectomy but not with late recurrence.^44^ This may be because preoperative NLR > 2.5 is positively associated with higher histological differentiation and vascular invasion rates of tumors.^45^

A 2018 study on postoperative NLR predicting prognosis and recurrence of HCC after resection showed that postoperative NLR ≥ 2.29 indicated poor prognosis, postoperative NLR ≥ 2.41 indicated early recurrence, and NLR ≥ 2.15 indicated late recurrence.^46^ Recent studies have found that postoperative NLR can better predict DFS after hepatectomy, and the prognostic model combining preoperative and postoperative NLR improved the prediction performance, which is better than the Barcelona Clinic Liver Cancer staging.^47^ Based on these studies, future studies on prognostic prediction models after hepatectomy should pay more attention to postoperative inflammatory indicators or the combination of multiple inflammatory indicator models.

Another inflammatory prognostic score that has attracted the attention of many researchers is the PLR. Studies have identified high preoperative PLR as an important prognostic marker after resection of various tumors, including of gastric cancer, pancreatic cancer, colorectal cancer, lung cancer, etc. In HCC, preoperative PLR ≥ 115 predicted poor OS and DFS for patients after hepatectomy.^48^ Kaida et al.^49^ confirmed that PLR was a good indicator to predict recurrence beyond the Milan criteria after liver resection for patients with HCC. Meanwhile, high PLR (≥150) levels were significantly associated with higher serum des‐γ‐carboxyprothrombin levels, larger tumor size, and poorer histological differentiation.

In addition, a previous study has reported that the LMR is an independent predictor of DFS and OS after HCC resection.^41^ Another retrospective study which recruited 1020 patients undergoing hepatectomy confirmed the superiority of LMR in predicting tumor recurrence in HCC patients after hepatectomy compared with other inflammation‐based markers.^50^ Low LMR (≤3.23) was positively associated with HCC recurrence.^50^

Recently, several studies have identified new inflammatory markers that predict prognosis in patients with hepatocellular carcinoma after hepatectomy. Iseda et al. reported that high lymphocyte‐to C‐reactive protein ratio (LCR ≥ 8400) was an independent predictor of RFS and OS in patients with HCC after hepatectomy, and was associated with the tumor microenvironment immune status.^51^ Iseda's another study found that preoperative albumin lymphocyte‐platelet‐C‐reactive protein (ALPC) index was associated with prognostic value in patients undergoing radical hepatectomy for hepatocellular carcinoma (HCC).^52^ It found that a high ALPC index was an important predictor of OS and RFS in patients undergoing surgical resection for liver cancer. This may be related to the expression of phosphorylation of antioxidant factor Nrf2 in tumors.^52^ A retrospective study from Japan found that the CRP‐albumin‐lymphocyte index (CALLY index) is a promising biomarker for predicting postoperative outcomes in HCC patients. The study found that patients with low CALLY index (≤5) had better survival and lower recurrence rates after hepatocellular carcinoma resection.^53^ All of these studies had certain limitations, including sample size, geography and other indicators.

**TABLE 1:** Calculation of prognostic indicators based on inflammation after hepatocellular carcinoma resection.

Inflammation based prognostic index	Score
Glasgow prognostic score (GPS)	
CRP ≤10 mg/L + albumin ≥35 g/L	0
CRP >10 mg/L + albumin <35 g/L	1
CRP >10 mg/L + albumin <35 g/L	2
mGPS	
CRP ≤10 mg/L	0
CRP >10 mg/L + Albumin ≥35 g/L	1
CRP >10 mg/L + Albumin <35 g/L	2
ALBI	
[log10 bilirubin (in lmol/L) × 0.66] + [albumin (in g/L) × −0.085] ≤−2.6	1
−2.6<[log10 bilirubin (in lmol/L) × 0.66] + [albumin (in g/L) × −0.085] ≤−1.39	2
[log10 bilirubin (in lmol/L) × 0.66] + [albumin (in g/L) × −0.085] >−1.39	3

**TABLE 2:** The cutoff values of prognostic indicators based on inflammation after hepatocellular carcinoma resection.

Inflammation based prognostic index	Cutoff
Preoperative NLR	2.81
Postoperative NLR	2.29
Preoperative PLR	115
LMR	3.23
LCR	8400
CALLY index	5

**FIGURE 1:**
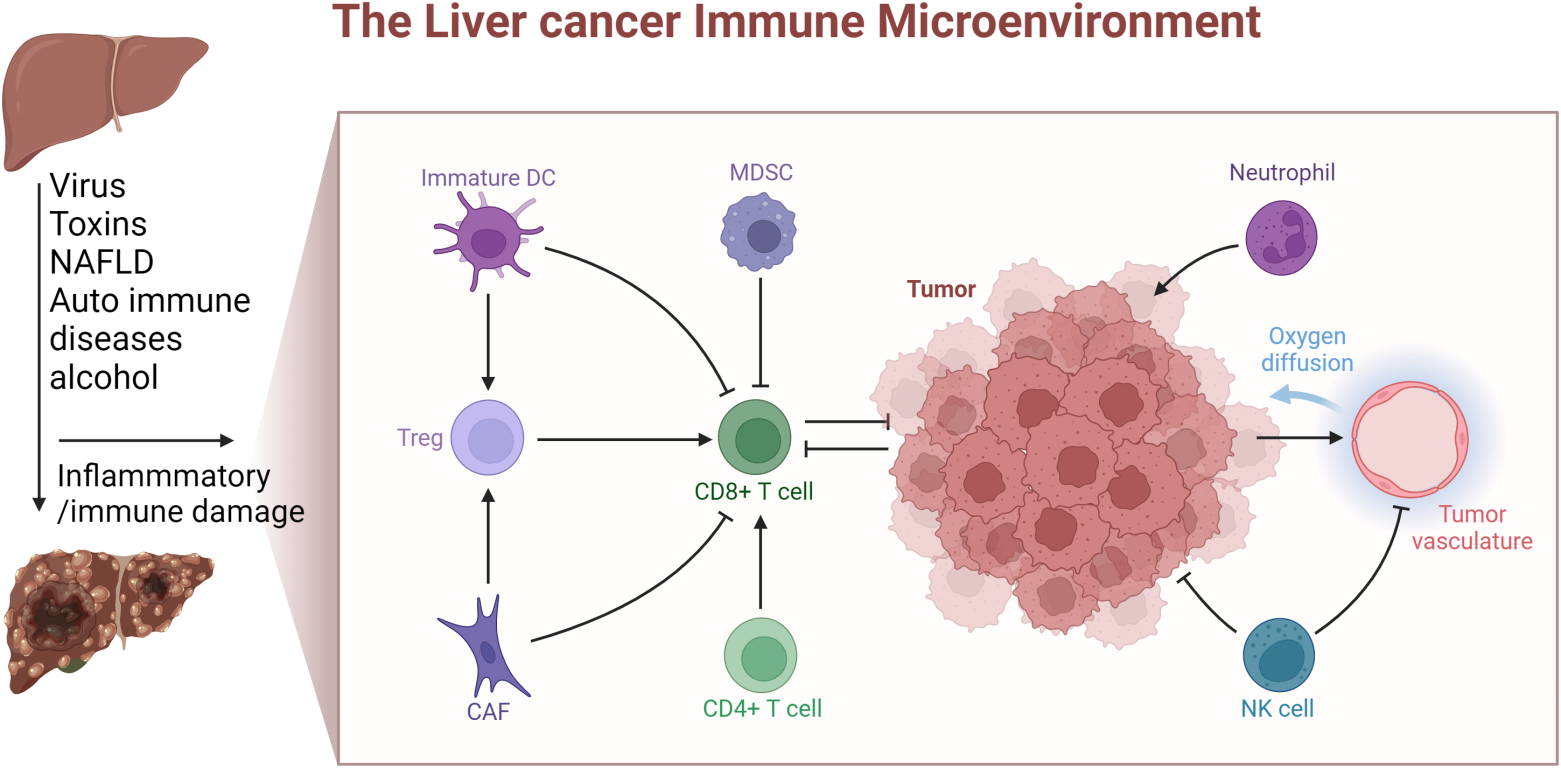
The liver cancer immune microenvironment.

All these studies suggest that inflammation plays an important role in tumor recurrence in patients after liver resection (Tables 1 and 2). Moreover, a small number of studies have explored the mechanism by which inflammation induces relapse. Still other studies have found that liver tissue damage after hepatectomy induces the release of inflammatory cells and inflammatory factors in body fluids to promote liver repair.^28^ These cytokines and inflammatory cells establish a self‐stimulating cycle, which subsequently causes the release of various mediators, such as vascular endothelial growth factor (VEGF), platelet‐derived growth factor, etc, which ultimately lead to tumor recurrence.^54^ Further research found that increase of the pro‐inflammatory cytokine tumor necrosis factor‐α in the peritumoral microenvironment upregulated the transcriptional regulator Snail to activate the NF‐κB pathway and promote tumor recurrence and metastasis.^55^ The tumor recurrence‐free survival (RFS) rate of patients after HCC resection was negatively correlated with the high expression of p65 and Snail. These results suggest that controlling hepatic inflammation and/or targeting NF‐κB‐mediated Snail expression may be potential therapeutic strategies to prevent HCC recurrence after hepatectomy.^55^

## 4 ROLE OF IMMUNE FACTORS IN RECURRENCE OF HCC AFTER CURATIVE RESECTION

In recent years, the advent of immune checkpoint blockers has led to significant progress towards treatment of unresectable HCC. At the same time, studies have found that for resectable HCC, the patient's immune activation and exhaustion play an important role in postoperative recurrence. Therefore, more and more studies have focused on describing immune infiltration to predict prognosis and recurrence after surgical treatment.

### 4.1 Cytotoxic T cells and Treg cells

Cytotoxic T lymphocytes, a subset of leukocytes, are specific T cells that secrete various cytokines and participate in immune function. They have a killing effect on some antigenic substances such as viruses and tumor cells and constitute an important defense line of antivirus and antitumor immunity with NK cells. Recent studies have demonstrated the importance of cytotoxic T lymphocytes on the recurrence of HCC after resection.^13^ Previous studies have shown that CD8+ T cells are closely associated with a lower recurrence rate and better prognosis after treatment for HCC.^56^

Gabrielson et al.^57^ found that the high density of CD3+ and CD8+ T cells in the intratumor and infiltrating margin of HCC, as well as the corresponding immune score, were significantly associated with a lower recurrence rate (*p* = 0.007) and longer RFS after HCC resection (*p* = 0.002). It does not depend on other predictive clinicopathological factors. Combined analysis showed that patients with higher CD3+ and CD8+ cell densities in one or both tumor regions (intratumor or infiltrating margin) had significantly lower HCC recurrence rates (*p* = 0.002). Positive PD‐L1 staining was associated with higher CD3+ and CD8+ density (*p* = 0.024 and *p* = 0.005, respectively), indicating a lower recurrence rate (*p* = 0.034) and prolonged RFS (*p* = 0.029).^57^ Another study found that tumor infiltrating CD4+ cells were also an independent predictor of DFS and OS after HCC resection.^58^

In contrast to cytotoxic T lymphocytes, which generally have inhibitory effects on tumor growth, Treg cells are thought to have a positive effect on tumor growth by inhibiting antitumor immune cells. Treg cells are an immunosuppressive subset of CD4+ T cells, and the forkhead box protein P3 (FoxP3) is its natural marker, which maintains immune homeostasis in immune tolerance and autoimmune control. Treg cells impair protective cancer immune surveillance. Numerous studies have found increased infiltration of Treg cells in tumor tissues of patients with HCC and considered Treg cells as a potential prognostic marker and target for therapeutic intervention.^59^ Increased numbers of FoxP3‐positive Treg cells have been shown to be an independent predictive factor of tumor recurrence after hepatectomy for HCC.^60^ The number of Treg cells was also reported to be positively correlated with preoperative serum AFP levels.^61^ Although a small number of studies have confirmed that Treg cells are associated with recurrence and poor prognosis after HCC resection, a large number of clinical studies are still needed for verification, and the underlying regulatory mechanism needs to be further explored.

In addition, liquid biopsy has the potential to serve as a biomarker for the detection, prognosis and monitoring of HCC,^62^ especially circulating tumor cells. But the use of liquid biopsies has its limitations. Before liquid biopsy can be recommended in the detection and clinical management of HCC, further research using standardized definition and methods of detection, quantification, and characterizing of CTCs; as well as standardized study design, patient selection, and outcome reporting will be required.

### 4.2 Macrophages

Macrophages are a major component of immune cell infiltration in almost all malignancies. Depending on the microenvironment of different types of tumors, macrophages undergo different forms of activation and polarization to play a tumor‐promoting or anti‐tumor role. In a retrospective study in patients after liver resection, Ding et al.^63^ found that intratumoral macrophage density and marginal macrophage density were inversely correlated with OS and DFS, while peritumoral macrophage density was not associated with survival. Multivariate Cox regression analysis revealed that intratumoral macrophage density was an independent prognostic factor for death and recurrence.^63^ However, another study had different results, in that the density of macrophages in the tumor was not associated with HCC recurrence.^64^ A meta‐analysis including five studies on macrophages and recurrence after hepatectomy found that the role of macrophages in recurrence after hepatectomy was inconclusive.^13^ This may be related to the different polarization of macrophages under different microenvironment regulation.

### 4.3 NK cells

NK cells, which make up 50% of the total number of hepatic lymphocytes, exert anti‐tumor effects by secreting granules containing lyases or triggering cell apoptosis. Studies have shown that NK cell dysfunction often leads to disease progression in multiple types of human solid tumors.^65^ In HCC, NK cells have a positive effect on OS after hepatectomy, but the effect on recurrence after hepatectomy is unknown.^49^ More clinical studies are needed to explore the relationship between NK cells and the prognosis of HCC treatment.

In summary, the number and complex interactions of tumor infiltrating immune cells can affect the survival rate of HCC hepatectomy (Figure 1). Current studies have confirmed that immunoeffector cells (CD3+ T and CD8+ T) and immunosuppressor cells (T‐regs macrophages, etc.) show considerable influence on postoperative tumor recurrence, and can be used as targets for immunoregulatory therapy. However, due to cell groups with smaller quantities in the tumor, it is difficult to serve as additional targets for antitumor treatment. Therefore, in order to more accurately predict the survival rate after HCC hepatectomy, more and more studies have begun to focus on the role of circulating immune cells, which will also be the focus of future research.

## 5 PREVENTION OF HCC AFTER HEPATECTOMPY

Due to the high 5‐year recurrence rate in patients with liver cancer after surgical resection, it is recommended to customize individualized adjuvant therapy after surgery. However, the current major guidelines do not recommend specific treatment options for preventive treatment after hepatectomy. In the last 10 years, a variety of clinical trials have focused on the efficacy of adjuvant therapy in preventing recurrence of HCC after hepatectomy, including antiviral therapy, molecular targeted therapy, immunotherapy, and traditional Chinese medicine therapy.

Studies have found that continuous and effective antiviral therapy can reduce recurrence and prolong OS and DFS in patients with hepatitis‐related HCC after surgery.^66^ Postoperative adjuvant transarterial chemoembolization can significantly reduce the intrahepatic recurrence rate and improve OS, especially in patients with high recurrence risk (tumor diameter >5 cm, microvascular invasion positive, or multi‐nodular tumors).^66^

The idea that adjuvant therapy with sorafenib can benefit patients with HCC after hepatectomy remains controversial.^66^ The STORM trial showed that sorafenib had no significant effect on RFS, recurrence time, or OS in patients after hepatectomy.^67^ Another European study also showed that adjuvant sorafenib did not have any substantial clinical benefit in terms of survival.^68^ In contrast, several recent retrospective studies in China have shown that sorafenib can reduce postoperative recurrence and prolong survival.^69^, ^70^ Therefore, more clinical studies are still needed to fully realize the maximal efficacy of molecular target drugs in postoperative adjuvant therapy. Current studies have shown that adjuvant adoptive immunotherapy can significantly improve the early (<3 years) clinical prognosis (recurrence rate and survival rate), but the long‐term (>5 years) efficacy needs further study.^71^ Moreover, many randomized controlled trials evaluating adjuvant immune checkpoint inhibitors after hepatectomy are ongoing. Recently, the famous IMbrave050 study released its Phase III clinical study data. IMbrave050 was a Phase III trial of atezolizumab plus bevacizumab in high‐risk hepatocellular carcinoma after curative resection or ablation. The results showed that combined blocking of PD‐L1/VEGF could significantly improve OS, PFS and response rate. The mechanism may be that concurrent PD‐L1 and VEGF inhibition may be effective in reducing HCC recurrence by creating a more immune‐favorable microenvironment, thereby enhancing anticancer immunity.^72^ In addition, a team from China found that Huaier (Trametes robiniophila Murr. extract) granules could reduce recurrence and prolong RFS after surgery.^73^ Based on these findings, it is necessary to customize individualized adjuvant therapy after hepatectomy.

## 6 CONCLUSIONS AND FUTURE PERSPECTIVES

By reviewing the relevant clinical studies on the recurrence of HCC after liver resection in the past 10 years, it was found that in addition to tumor factors, the systemic inflammatory response and a patient's immune disorder can accelerate the recurrence of HCC. At present, various inflammatory and immune scoring systems have been used to predict the recurrence and prognosis of HCC after surgery, but these studies have limitations. However, the latest research proposes the use of nomogram mode to incorporate various scoring systems to improve the value of predicting recurrence and prognosis of HCC. Moreover, considering the high recurrence rate after HCC resection, we also recommend that clinicians customize individualized adjuvant treatment plans according to the patients' conditions after surgery to reduce the recurrence rate and prolong RFS and OS.

## AUTHOR CONTRIBUTIONS

**Sha she:** Writing – original draft (equal); writing – review and editing (equal). **Jinzhi Shi:** Writing – original draft (equal); writing – review and editing (equal). **Jiling Zhu:** Data curation (equal); formal analysis (equal). **Fan Yang:** Data curation (equal); formal analysis (equal). **Jia Yu:** Conceptualization (equal); funding acquisition (equal); project administration (equal); writing – review and editing (equal). **Kai Dai:** Conceptualization (equal); funding acquisition (equal); project administration (equal); writing – review and editing (equal).

## FUNDING INFORMATION

This work supported by the National Natural Science Foundation of China, No. 81972673 and the Interdisciplinary Innovative Talents Foundation from Renmin Hospital of Wuhan University (JCRCFZ‐2022‐028).

## CONFLICT OF INTEREST STATEMENT

The authors declare no conflict of interest.

## Data Availability

Data sharing is not applicable to this article as no new data were created or analyzed in this study.
